# Giant Dilatation of the Right Coronary Aortic Bulb with Compression of the Right Ventricular Outflow Tract Mimicking a Ventricular Septal Defect: Diagnostic workup Using Echocardiography, Heart Catheterization, and Cardiac Computed Tomography

**DOI:** 10.1155/2012/524526

**Published:** 2012-08-16

**Authors:** Nina P. Hofmann, Hassan Abdel-Aty, Stefan Siebert, Hugo A. Katus, Grigorios Korosoglou

**Affiliations:** ^1^Department of Cardiology, Heidelberg University, Im Neuenheimer Feld 410, 69120 Heidelberg, Germany; ^2^Department of Radiology, Heidelberg University, Im Neuenheimer Feld 410, 69120 Heidelberg, Germany

## Abstract

Annuloaortic ectasia is a relatively rare diagnosis. Herein, we report an unusual case of an annuloaortic ectasia with asymmetric dilatation of the right coronary bulb mimicking a membranous ventricular septal defect (VSD) with Eisenmenger reaction by transthoracic echocardiography. Aortic angiography showed a dilated aortic root and moderate aortic regurgitation. Right cardiac catheterization, on the other hand, exhibited normal pulmonary artery blood pressure and normal pulmonary resistance, whereas normal venous gas values were measured throughout the caval vein and the right atrium, excluding relevant left-right shunting. Further diagnostic workup by cardiac computed tomography angiography (CCTA) unambiguously illustrated the asymmetric geometry of the ectatic aortic cusp and root causing compression of the right heart and of the right ventricular (RV) outflow tract. After review of echocardiographic acquisitions, the blood flow detected between the left and right ventricles (mimicking VSD) was interpreted as turbulent inflow from the left ventricle into the ectatic right coronary cusp. Furthermore, elevated pulmonary artery blood pressure measured by echocardiography was attributed to “functional pulmonary stenosis” due to compression of the RV outflow tract by the aorta, as demonstrated by CCTA.

## 1. Case Report

**Figure 1:**
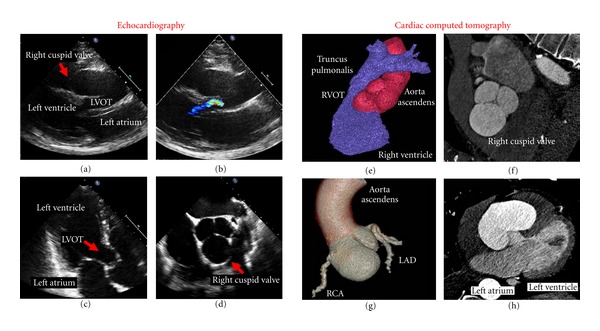
Comparison of transthoracic echocardiography and cardiac computed tomography angiography (CCTA). Short parasternal axis with and without aortic outflow tract (a) and (b), two-chamber view with left ventricular outflow tract (c), and top view with tricuspid aortic valve (d) and (f). Compression of the right ventricular outflow tract (e), 3D reconstruction of the aortic root (g), and four-chamber view (h) in CCTA. (LVOT: left ventricle outflow tract, RVOT: right ventricle outflow tract, LAD: left anterior descending, RCA: right coronary artery.)

A 67-year-old male patient with history of arterial hypertension, atrial fibrillation, and cigarette smoking (75 pack/years) was admitted to our department due to exertional dyspnoea. Transthoracic echocardiography indicated a VSD (Figure 1(a)) with blood flow between the left and RV by colour Doppler (Figure 1(b)) possibly causing elevated pulmonary pressure (RV systolic pressure of 50 mmHg). Furthermore, an ectasia of the aortic bulb was observed in the apical view (Figure 1(c)), which was confirmed by transesophageal echocardiography (TEE, Figure 1(d), aortic root diameter of 65 mm).

**Figure 2:**
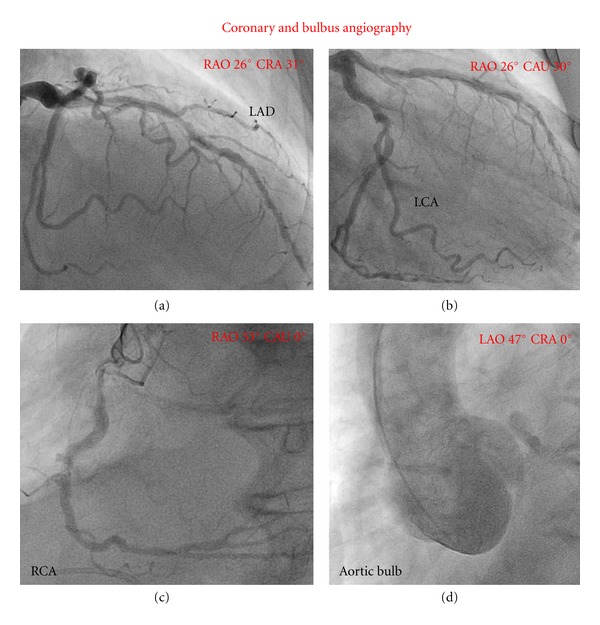
Severe coronary three-vessel disease (a)–(c) and annuloaortic ectasia (d) revealed in cardiac catheter. (LAD: left anterior descending, LCX: left circumflex artery, RCA: right coronary artery.)

Due to the suspected VSD and secondary pulmonary hypertension, the patient was scheduled for right cardiac catheterization and coronary angiography. Hereby, unexpectedly normal pulmonary artery pressure (systolic pulmonary artery pressure of 30 mmHg and normal pulmonary resistance) was measured during right heart catheterization, whereas coronary angiography revealed a severe three-vessel disease (Figures 2(a)–2(c)). Furthermore, aortic angiography exhibited moderate aortic regurgitation associated with an ectatic aortic root and an aneurysm of the ascending aorta (diameter of 5.3 cm, Figure 2(d)).

The patient was subsequently scheduled for aortocoronary bypass graft surgery and aortic root and valve replacement. A 256-slice cardiac computed tomography angiography (CCTA) was performed (Philips Best, Netherlands Healthcare) for additional imaging of the aortic root prior to surgery. Hereby, an extremely asymmetric dilatation of the right coronary aortic cusp was observed, causing severe compression of the RV and of the RV outflow tract (Figures 1(e)–1(h)). A maximal diameter of 7.3 cm was measured at the level of the aortic bulb on three-dimensional (3D) reconstructions. After review of echocardiographic acquisitions, the blood flow detected between the LV base and RV (mimicking VSD) was interpreted as turbulent inflow from the left ventricle (LV) into the ectatic right coronary cusp, whereas the elevated pulmonary blood pressure was attributed to compression of the RV outflow tract, as demonstrated by CCTA. 

Subsequently, the patient underwent heart surgery for aortocoronary bypass implantation (A. mammaria interna to the first diagonal and V. saphena magna to the first marginal; LAD and RCA were not suitable for connection) and a modified Bentall procedure for aortic valve, root, and ascending aorta replacement (involving implantation of a mechanical aortic valve).

## 2. Discussion

Dilatation of the aortic root and of the ascending aorta is often associated with a bicuspid aortic valve with concomitant aortic regurgitation [1]. Other causes include Marfan, Ehlers-Danlos, and Loeys-Dietz syndromes [2–4]. The most feared complication is the dissection or rupture of the aortic root. Therefore, annual imaging is recommended in case of expected progressive dilatation (diameter > 40 mm), whereas prophylactic surgical intervention is deemed appropriate when bicuspid aortic valves associated with aortic dilatation (diameter > 55 mm) are present [5].

On the other hand, when a dilated aortic root is detected in older patients with a tricuspid aortic valve, an accurate cardiovascular survey that includes the entire aorta is necessary, due to the suspected systemic nature of the aortic dilatation in this cohort [6]. In such cases, combined surgical aortic root and valve replacement are recommended [7]. 

The method of choice for identification and characterization of VSD is colour Doppler, TEE, and 3D echocardiography. Other diagnostic approaches include clinical examination (pan-systolic murmur, diastolic rumble at the apex), electrocardiography (signs of LV and RV hypertrophy), cardiac catheterization (pulmonary resistance), and magnetic resonance imaging (MRI, pulmonary-to-systolic flow ratio) [8].

Pulmonary hypertension, on the other hand, is mostly examined during right heart catheterization for assessment of the RV pressure, pulmonary artery pressure, and pulmonary capillary pressure (wedge). Patient history, physical examination, chest radiography, electrocardiography, transthoracic echocardiography, cardiac computed tomography, and MRI are used for excluding possible causes of pulmonary hypertension as congestive heart failure, lung diseases, and pulmonary embolism. Rare causes include congenital or familial diseases, connective tissue disorders, human immunodeficiency virus infection, portal hypertension, chronic haemolysis, and drugs [9]. In our case, obstruction of the RVOT caused “functional pulmonary stenosis.” This possibly resulted in an overestimation of the PA pressure as measured by echo Doppler. Probably a slow pull-back with an end-hole catheter during right heart catheterization may have revealed a pressure gradient in the RV, confirming functional obstruction. Unfortunately, and because right heart catheterization was performed prior to CCTA, this data is not available.

Our patient presented with exertional dyspnoea and suspected VSD with secondary pulmonary hypertension, as initially diagnosed by transthoracic echocardiography. Heart catheterization, however, revealed normal pressures in the pulmonary artery and in the RV, so that the initial diagnosis established by echocardiography was considered unlikely. 

In contrast to CCTA, the assessment of the aortic root in the catheter laboratory is based on two-dimensional images and may therefore underestimate the maximal diameter of the aortic root, as in the case presented herein. In contrary to echocardiography, TEE, and invasive bulbus angiography, CCTA unambiguously detected the giant dilatation of the right coronary bulb causing functional obstruction of the RV outflow tract. Thus, CCTA helped to understand why a VSD was misdiagnosed by echocardiography, and established the final diagnosis. Furthermore, the exact diameter, form, and neighbouring anatomical structures of the annuloaortic ectasia could be delineated on 3D reconstructions.
